# Supplementary material for: Vitamin D status in chronic fatigue syndrome/myalgic encephalomyelitis: a cohort study from the North-West of England
Source: BMJ Open. 2017 Nov 8;7(11):e015296. doi: 10.1136/bmjopen-2016-015296 (PMC5695299; doi:10.1136/bmjopen-2016-015296)
Supplement: Author's manuscript [file bmjopen-2016-015296.draft_revisions.pdf]

# Vitamin D status in Chronic Fatigue Syndrome/Myalgic Encephalomyelitis: a cohort study

| | |
|---|---|
| Journal: | *BMJ Open* |
| Manuscript ID | bmjopen-2016-015296 |
| Article Type: | Research |
| Date Submitted by the Author: | 08-Dec-2016 |
| Complete List of Authors: | Earl, Kate ; MRC-Arthritis Research UK Centre for Integrated Research into Musculoskeletal Ageing, Department of Musculoskeletal Biology, Institute of Ageing and Chronic Disease, University of Liverpool<br>Sakellariou, Giorgos ; MRC-Arthritis Research UK Centre for Integrated Research into Musculoskeletal Ageing, Department of Musculoskeletal Biology, Institute of Ageing and Chronic Disease, University of Liverpool; GeneFirst Ltd, Culham Science Centre, Abingdon, ,<br>Sinclair, Melanie; MRC-Arthritis Research UK Centre for Integrated Research into Musculoskeletal Ageing, Department of Musculoskeletal Biology, Institute of Ageing and Chronic Disease, University of Liverpool<br>Fenech, Manuel ; MRC-Arthritis Research UK Centre for Integrated Research into Musculoskeletal Ageing, Department of Musculoskeletal Biology, Institute of Ageing and Chronic Disease, University of Liverpool; 3Tropical and Infectious Disease Unit, Royal Liverpool University Hospital,<br>Croden, Fiona ; Human Appetite Research Unit, School of Psychology, University of Leeds,<br>Owens, Daniel ; Research Institute for Sport and Exercise Science, Liverpool John Moores University,<br>Tang, Jonathan ; University of East Anglia Faculty of Medicine and Health Sciences, Department of Medicine Norwich Medical School, Faculty of Medicine and Health Sciences, University of East Anglia, Norwich Research Park,<br>Miller, Alastair; Royal Liverpool and Broadgreen University Hospitals<br>Lawton, Clare ; Human Appetite Research Unit, School of Psychology, University of Leeds,<br>Dye, Louise ; Human Appetite Research Unit, School of Psychology, University of Leeds,<br>Close, Graeme ; Research Institute for Sport and Exercise Science, Liverpool John Moores University,<br>Fraser, William ; Department of Medicine Norwich Medical School, Faculty of Medicine and Health Sciences, University of East Anglia, Norwich Research Park,<br>McArdle, Anne ; MRC-Arthritis Research UK Centre for Integrated Research into Musculoskeletal Ageing, Department of Musculoskeletal Biology, Institute of Ageing and Chronic Disease, University of Liverpool<br>Beadsworth, Michael; Royal Liverpool University Hosp, |
| <b>Primary Subject Heading</b>: | Nutrition and metabolism |
| Secondary Subject Heading: | Pathology |

| Keywords: | Chronic fatigue syndrome, Myalgic encephalomyelitis, Vitamin D, Supplements, CFS/ME |
|---|---|
| | |

SCHOLARONE™
Manuscripts

For peer review only

* . Total serum 25[OH]D concentration in patients with CFS/ME and healthy controls (HC) who reported no intake of vitamin D supplements. Each data point refers to an individual participant. Solid black and grey bars represent the mean ± SD.

For peer review only

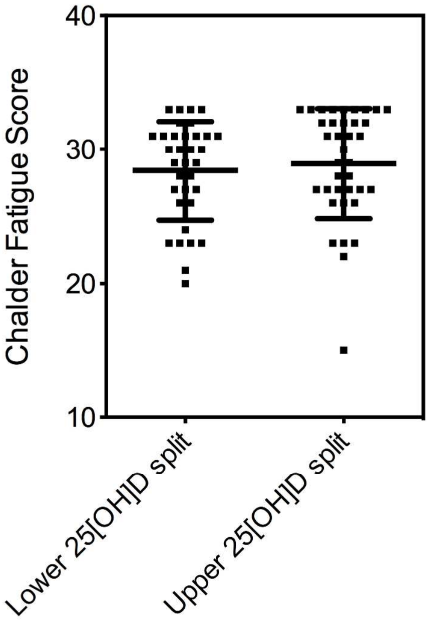


+ . Chalder fatigue scores in patients with CFS/ME. 25[OH]D values from the CFS/ME group were ordered from lowest to highest and split equally into an upper and lower range. Each data point refers to an individual participant. Solid black and grey bars represent the mean ± SD.

For peer review only

,

, $ Baseline characteristics of study participants. Data are presented as mean ± SD unless otherwise stated.

| | - | |
|---|---|---|
| . % & | 58 | 66 |
| % & | 36 | 26 |
| % & | 89 | 91 |
| %/ & | 33 ± 10 | 40 ± 10 |
| ( %0 1& | 24.4 ± 3.4 | 26.0 ± 4.2 |
| % & | N/A | 36 ± 4.8 |
| 2 | 94 | 92 |

, * . Dietary/supplement assessment of average Vitamin D intake by patients with CFS/ME compared with HC individuals. Data are presented as mean ± SD.

| | - | | |
|---|---|---|---|
| # # 0 3 %4 & | 2.9 ± 2.1 | 2.4 ± 1.6 | 0.6 |
| # # 0 3 %4 & | 0.6 ± 1.7 | 4.9 ± 9.7 | 0.05 |

For peer review only

!

!
" #$% # & '( (
' ) * + ,* -* .
/
/ 0 1 '( %& **23** 2 %3 432
% 50 )* , 66 * #* 7 /
" ) 1
8 '( 9& **114** 2 %(:4'(
9 1 8;* . 1#* ! ;1 7 "
+ '( '& **45** ' <<(4<2
$ " -* = >* 8 "4 54?* 7 - @8'3+ A ' '
" 6
1 ) 8 4 8 '( '& **303** 9 %:24
9($
2 7 # - / / 0 "
+ ! ) '(($& **330** 39:( $'94'2
3 + " .* * A +!* / /
" A B ! 6
'( %& **10**
< + " .* ! .+* ; ;-* 7 0
4 " A "
'( 9& **65** ' %94%3
: 50 )* 18* 8 * 1 + / 7
" ! - * - ; 1 ) 8
! 6 '( $ C ((%3$ '( $
( + 4 ;1* .* 5 / )* ; '$ 4 A /
0 6 0 4 A 6 /
/ DE 2( 1 ) '((9& **80** % 3$'4$<
# 8* - 6 ?* 56 ?* 1 6 0 '$4 A /
/ * / * / 4
6 58-1 , 5 '(($& **16** 9'$4
%
' 1* ; 0 ?#* + * / 7 7
1 1 , / ! 5A /
" ! ) B ! 6 '( %& **98** % $(:4
%
% ! !* ! / F* + G 6 6
0 /
" 6 F '((3& **28** 9 9$242'
9 , -* " ! H - 1 /
6 7 '( $& **7** ( <'$ 42(
$ , " ;* ! * ?) . 6 0
/ 1
6 ,
* - # ! '(((& **1** 9 '<%4
2
2 8 F1* F ; 7 * / 0
/ '( (& **9** ' :9 4$$

For peer review only

3 1 1* . !* . GG -* 0 /
" I 1 / 0
1 / 0 '( '& **12** ' '34%2
< = 6 ?* ) " - / 6 0 " 0 /
, ) ! ::3& **167** 2 9%$4%:
: + " / G * 1 6 #* ) " !* '$4 A 7 F /
- / / ) 7 - '((: &**79** 9 '$(4$9
'( F F* ! " / 7* *
1 - / '((:& **8** 9 '<34:
' + " #* )* ? G 1* 1 " 0 "
+ + / '( (& **24** 3 '(:4 3
" +1 ! + 1 ! . !
! 0006 '( $ 000 6
'% #* ! ;* # 6 -* /
6 0 / 6 8 '( (& **5** 3 $$$
'9 - !* 6 -* ; * 8 /
, * ? 1 / ! '((%& **163** % $%(4%2
'$ ; +, 1 '$4; A / *'$4
A / / -
1 '( '& **38** ':4J
'2 + !4F* ; ? * - 6 * . 1 6 0 7
7 * 8 0 * - " 1 56 / )
'( %& **2013** 3$ % (4 (
'3 F +* + 8* 1* F 0 / '$4 A /
6 0
6C 0 6 0
6 '((2& **49** ' '<934$'
'< )8* # * . 8 '$4; A / F / - "
; 1 @ , ; '((<& **52** $ <'<4%'
': ; 6 !+* # * + C F 0 / '$4 A / '$4
5; 4 0 - 0 * *
) + ! + '( (& **121** 4' 92343(
%( ! G ;* 1* ) * * 7 1
1 F - / 0 '( 2& **8** 9 '%2
% 50 )* . ) * + ,)* ; 7
1 ! A '( 2
%' ) ! * # #* @* 6 4 / 6 '$4
A / 6 0 6 '$4
A / / C 6 /
'( 9& **74** % 334<%

For peer review only

STROBE Statement—checklist of items that should be included in reports of observational studies

| | Item No | Recommendation | Page No |
|---|---|---|---|
| **Title and abstract** | 1 | (*a*) Indicate the study's design with a commonly used term in the title or the abstract | 1 |
| | | (*b*) Provide in the abstract an informative and balanced summary of what was done and what was found | 2 |
| **Introduction** | | | |
| Background/rationale | 2 | Explain the scientific background and rationale for the investigation being reported | 3 |
| Objectives | 3 | State specific objectives, including any prespecified hypotheses | 4 |
| **Methods** | | | |
| Study design | 4 | Present key elements of study design early in the paper | 4 |
| Setting | 5 | Describe the setting, locations, and relevant dates, including periods of recruitment, exposure, follow-up, and data collection | 4 |
| Participants | 6 | (*a*) *Cohort study*—Give the eligibility criteria, and the sources and methods of selection of participants. Describe methods of follow-up | N/A |
| | | *Case-control study*—Give the eligibility criteria, and the sources and methods of case ascertainment and control selection. Give the rationale for the choice of cases and controls | N/A |
| | | *Cross-sectional study*—Give the eligibility criteria, and the sources and methods of selection of participants | 4 |
| | | (*b*) *Cohort study*—For matched studies, give matching criteria and number of exposed and unexposed | N/A |
| | | *Case-control study*—For matched studies, give matching criteria and the number of controls per case | N/A |
| Variables | 7 | Clearly define all outcomes, exposures, predictors, potential confounders, and effect modifiers. Give diagnostic criteria, if applicable | 4 |
| Data sources/ measurement | 8* | For each variable of interest, give sources of data and details of methods of assessment (measurement). Describe comparability of assessment methods if there is more than one group | 4 & 5 |
| Bias | 9 | Describe any efforts to address potential sources of bias | 4 & 5 |
| Study size | 10 | Explain how the study size was arrived at | 4 |
| Quantitative variables | 11 | Explain how quantitative variables were handled in the analyses. If applicable, describe which groupings were chosen and why | 6 |
| Statistical methods | 12 | (*a*) Describe all statistical methods, including those used to control for confounding | 6 |
| | | (*b*) Describe any methods used to examine subgroups and interactions | 6 |
| | | (*c*) Explain how missing data were addressed | N/A |
| | | (*d*) *Cohort study*—If applicable, explain how loss to follow-up was addressed | N/A |
| | | *Case-control study*—If applicable, explain how matching of cases and controls was addressed | 4 |
| | | *Cross-sectional study*—If applicable, describe analytical methods taking account of sampling strategy | 6 |

For peer review only

| | | |
|---|---|---|
| | (*e*) Describe any sensitivity analyses | 6 |

Continued on next page

For peer review only



For peer review only - http://bmjopen.bmj.com/site/about/guidelines.xhtml

| | | | |
|---|---|---|---|
| **Results** | | | |
| Participants | 13* | (a) Report numbers of individuals at each stage of study—eg numbers potentially eligible, examined for eligibility, confirmed eligible, included in the study, completing follow-up, and analysed | 6 |
| | | (b) Give reasons for non-participation at each stage | N/A |
| | | (c) Consider use of a flow diagram | N/A |
| Descriptive data | 14* | (a) Give characteristics of study participants (eg demographic, clinical, social) and information on exposures and potential confounders | 6 |
| | | (b) Indicate number of participants with missing data for each variable of interest | 7 |
| | | (c) *Cohort study*—Summarise follow-up time (eg, average and total amount) | N/A |
| Outcome data | 15* | *Cohort study*—Report numbers of outcome events or summary measures over time | |
| | | *Case-control study*—Report numbers in each exposure category, or summary measures of exposure | N/A |
| | | *Cross-sectional study*—Report numbers of outcome events or summary measures | 7 |
| Main results | 16 | (*a*) Give unadjusted estimates and, if applicable, confounder-adjusted estimates and their precision (eg, 95% confidence interval). Make clear which confounders were adjusted for and why they were included | 6 & 7 |
| | | (*b*) Report category boundaries when continuous variables were categorized | 7 |
| | | (*c*) If relevant, consider translating estimates of relative risk into absolute risk for a meaningful time period | N/A |
| Other analyses | 17 | Report other analyses done—eg analyses of subgroups and interactions, and sensitivity analyses | 7 |
| **Discussion** | | | |
| Key results | 18 | Summarise key results with reference to study objectives | 8 |
| Limitations | 19 | Discuss limitations of the study, taking into account sources of potential bias or imprecision. Discuss both direction and magnitude of any potential bias | 9 |
| Interpretation | 20 | Give a cautious overall interpretation of results considering objectives, limitations, multiplicity of analyses, results from similar studies, and other relevant evidence | 8&9 |
| Generalisability | 21 | Discuss the generalisability (external validity) of the study results | 9 |
| **Other information** | | | |
| Funding | 22 | Give the source of funding and the role of the funders for the present study and, if applicable, for the original study on which the present article is based | 6 &10 |

*Give information separately for cases and controls in case-control studies and, if applicable, for exposed and unexposed groups in cohort and cross-sectional studies.

**Note:** An Explanation and Elaboration article discusses each checklist item and gives methodological background and published examples of transparent reporting. The STROBE checklist is best used in conjunction with this article (freely available on the Web sites of PLoS Medicine at http://www.plosmedicine.org/, Annals of Internal Medicine at http://www.annals.org/, and Epidemiology at http://www.epidem.com/). Information on the STROBE Initiative is available at www.strobe-statement.org.

For peer review only

BMJ Open

# Vitamin D status in Chronic Fatigue Syndrome/Myalgic Encephalomyelitis: a cohort study from the North-West of England

| | |
|---|---|
| Journal: | *BMJ Open* |
| Manuscript ID | bmjopen-2016-015296.R1 |
| Article Type: | Research |
| Date Submitted by the Author: | 25-Apr-2017 |
| Complete List of Authors: | Earl, Kate ; MRC-Arthritis Research UK Centre for Integrated Research into Musculoskeletal Ageing, Department of Musculoskeletal Biology, Institute of Ageing and Chronic Disease, University of Liverpool<br>Sakellariou, Giorgos ; MRC-Arthritis Research UK Centre for Integrated Research into Musculoskeletal Ageing, Department of Musculoskeletal Biology, Institute of Ageing and Chronic Disease, University of Liverpool; GeneFirst Ltd, Culham Science Centre, Abingdon, ,<br>Sinclair, Melanie; MRC-Arthritis Research UK Centre for Integrated Research into Musculoskeletal Ageing, Department of Musculoskeletal Biology, Institute of Ageing and Chronic Disease, University of Liverpool<br>Fenech, Manuel ; MRC-Arthritis Research UK Centre for Integrated Research into Musculoskeletal Ageing, Department of Musculoskeletal Biology, Institute of Ageing and Chronic Disease, University of Liverpool; 3Tropical and Infectious Disease Unit, Royal Liverpool University Hospital,<br>Croden, Fiona ; Human Appetite Research Unit, School of Psychology, University of Leeds,<br>Owens, Daniel ; Research Institute for Sport and Exercise Science, Liverpool John Moores University,<br>Tang, Jonathan ; University of East Anglia Faculty of Medicine and Health Sciences, Department of Medicine Norwich Medical School, Faculty of Medicine and Health Sciences, University of East Anglia, Norwich Research Park,<br>Miller, Alastair; Royal Liverpool and Broadgreen University Hospitals<br>Lawton, Clare ; Human Appetite Research Unit, School of Psychology, University of Leeds,<br>Dye, Louise ; Human Appetite Research Unit, School of Psychology, University of Leeds,<br>Close, Graeme ; Research Institute for Sport and Exercise Science, Liverpool John Moores University,<br>Fraser, William ; Department of Medicine Norwich Medical School, Faculty of Medicine and Health Sciences, University of East Anglia, Norwich Research Park,<br>McArdle, Anne ; MRC-Arthritis Research UK Centre for Integrated Research into Musculoskeletal Ageing, Department of Musculoskeletal Biology, Institute of Ageing and Chronic Disease, University of Liverpool<br>Beadsworth, Michael; Royal Liverpool University Hosp, |
| <b>Primary Subject Heading</b>: | Nutrition and metabolism |

| Secondary Subject Heading: | Pathology |
| --- | --- |
| Keywords: | Chronic fatigue syndrome, Myalgic encephalomyelitis, Vitamin D, Supplements, CFS/ME |
| | |

SCHOLARONE™ Manuscripts

For peer review only

Research Article BMJ Open

# Vitamin D status in Chronic Fatigue Syndrome/Myalgic Encephalomyelitis: a cohort study from the North-West of England

Kate E. Earl[1], Giorgos K. Sakellariou[1,2], Melanie Sinclair[1], Manuel Fenech[1,3], Fiona Croden[4], Daniel J. Owens[5], Jonathan Tang[6], Alastair Miller[3], Clare Lawton[4], Louise Dye[4], Graeme L. Close[5], William D. Fraser[6], Anne McArdle[1] and Michael B J. Beadsworth[1,3]

**Affiliations**

[1]MRC-Arthritis Research UK Centre for Integrated Research into Musculoskeletal Ageing, Department of Musculoskeletal Biology, Institute of Ageing and Chronic Disease, University of Liverpool, UK. [2]Current address GeneFirst Ltd, Culham Science Centre, Abingdon, Oxfordshire, OX14 3DB, UK. [3]Tropical and Infectious Disease Unit, Royal Liverpool University Hospital, Liverpool, UK. [4]Human Appetite Research Unit, School of Psychology, University of Leeds, Leeds, UK. [5]Research Institute for Sport and Exercise Science, Liverpool John Moores University, Liverpool, UK. [6]Department of Medicine Norwich Medical School, Faculty of Medicine and Health Sciences, University of East Anglia, Norwich Research Park, Norwich, UK.

**Corresponding Author:**

Dr Michael BJ Beadsworth,

Consultant Infectious Diseases, Tropical Medicine, and Clinical Pharmacology,

Royal Liverpool University Hospital,

Liverpool, L7 8XP, U.K,

Tel: (44) 151 706 3836,

Fax: (44) 151 706 5944,

E-mail: Mike.Beadsworth@rlbuht.nhs.uk

For peer review only

**Abstract**

*Objective*: Severe vitamin D deficiency is a recognised cause of skeletal muscle fatigue and myopathy. The aim of this study was to examine whether Chronic Fatigue Syndrome / Myalgic Encephalomyelitis (CFS/ME) is associated with altered circulating vitamin D metabolites.

*Design*: Cohort study.

*Setting*: UK university hospital, recruiting from April 2014 to April 2015.

*Participants*: 92 patients with CFS/ME and 94 age-matched healthy controls.

*Main outcome measures:* The presence of a significant association between CFS/ME, fatigue and vitamin D measures.

*Results:* No evidence of a deficiency in serum total 25[OH] vitamin D (25[OH]$D_2$ and 25[OH]$D_3$ metabolites) was evident in individuals with CFS/ME. Liquid chromatography tandem mass spectrometry (LC-MS/MS) analysis revealed that total 25[OH]D was significantly higher ($p = 0.001$) in serum of CFS/ME patients compared with healthy controls (60.2 and 47.3nmol/L respectively). Analysis of food/supplement diaries with WinDiets revealed that the higher total 25[OH] vitamin D concentrations observed in the CFS/ME group were associated with increased vitamin D intake through use of supplements compared with the control group. Analysis of Chalder Fatigue Questionnaire data revealed no association between perceived fatigue and vitamin D levels.

*Conclusions:* Low serum concentrations of total 25 [OH]D do not appear to be a contributing factor to the level of fatigue of CFS/ME.

**Strengths and limitations of this study**

- This is the first cross-sectional analysis of vitamin D status to be determined in patients with CFS/ME.
- Patients were referred through a single NHS CFS services unit at the Royal Liverpool and Broadgreen University NHS trust and diagnosed with CFS/ME using the NICE guidelines ensuring diagnostic uniformity.
- Serum 25-hydroxyvitamin D (25-OHD), the main marker of vitamin D status was determined using liquid chromatography tandem mass spectrometry, comprehensive dietary/supplement analyses were undertaken to support vitamin D serum data.

For peer review only

- Recruitment included a broad spectrum of ambulatory patients with CFS/ME, age and sex matched to health controls but excluded the severest, non ambulatory CFS/ME patients.

## Introduction

Chronic Fatigue Syndrome (CFS), also referred to as Myalgic Encephalomyelitis (ME) is a debilitating and complex disease characterised by prolonged and disabling fatigue [1]. Patients with CFS/ME suffer from a range of symptoms including; headache, painful lymph nodes, sore throat, difficulty in sleeping or insomnia, poor cognitive function, muscle/joint pain and post-exertional malaise. CFS/ME can affect individuals of all ages, races and socioeconomic status. Prevalence of the disease has been estimated between 0.2 – 1% [2-4] . Despite the severity of CFS/ME and the prevalence of the disease, the aetiology and pathophysiology of CFS/ME is yet to be determined due to a lack of robust evidence within current literature to confirm any specific biological abnormalities. Current areas of investigation include infection, endocrine dysfunction, autonomic nervous system imbalance and altered immunity. Vitamin D regulation has become a leading focus of health professionals following its implication in health and disease [5-7]. Vitamin D metabolites are regarded as vital endocrine regulators of bone health via their role in calcium and phosphate homeostasis [8 9]. Evidence has shown that vitamin D deficiency (25 [OH]D <30nmol/L)[10] is associated with numerous adverse health outcomes, including risk of rickets in children or osteomalacia in adults, increased risk of fractures, falls, infectious disease, type 1 and type 2 diabetes, cancer, autoimmune disease, multiple sclerosis, hypertension and heart disease[11]. Since the identification of the vitamin D receptor (VDR) in almost all tissues of the human body including skeletal muscle [12], evidence suggests that vitamin D metabolites are also implicated in skeletal muscle function and repair [13-16]. Clinical trials undertaken in geriatric and clinical populations [13 17 18] have established a potential link between vitamin D-sufficiency, as assessed by circulating total 25 hydroxy vitamin D (25 [OH] D) and physical performance including measures such as balance, gait speed, handgrip and lower limb strength, highlighting the role of vitamin D status in muscle function. In support of these findings, longitudinal studies undertaken in vitamin D-deficient adults showed that correcting vitamin D deficiency with use of dietary supplement interventions resulted in improved muscle mitochondrial function, associated with an improvement in symptoms of myopathy and fatigue [19]. The potential importance of vitamin D signalling is also highlighted by the ability of vitamin D signalling to modulate the activation of nuclear factor kappa B (NFκB), a pleiotropic transcription factor involved in many biological and pathological processes

For peer review only

including inflammation and immunity [20]. Indeed, 1,25 $[OH]_2D$ is also proposed to be a potent regulator of innate and acquired immunity [21].

Due to the pleiotropic functions of vitamin D metabolites, the sterols have been examined in the context of disease and have consequently been correlated with a number of pathologies including tuberculosis, motor neurone and autoimmune diseases [22-24] and in clinical populations including patients with osteomalacia, who display skeletal muscle myopathy and weakness that is responsive to vitamin D supplementation [25]. Despite non-scientific media interest, few data exist examining circulating concentration of vitamin D metabolites in patients with CFS/ME. There has been only one retrospective study that suggests that patients with CFS/ME may experience suboptimal concentrations of circulating 25[OH]D [26], hence the assumption that low circulating 25 [OH]D concentrations in CFS/ME may be responsible for the altered immune profiles [27 28] and fatigue. The objective of the current study was to determine whether vitamin D status, as assessed by circulating 25[OH]D, is a contributing factor to the pathophysiology of CFS/ME. To meet this objective, we investigated whether CFS/ME was associated with altered serum concentrations of vitamin D metabolites.

## Methods

### Recruitment

Male and female patients with CFS/ME (n=92) were recruited through the regional NHS CFS services based in Liverpool at the Royal Liverpool and Broadgreen University Hospital Trust (RLUBHT), Merseyside, UK. All patients were newly diagnosed by clinicians in accordance with the recommended NICE guidelines (2007) [29] and covered a wide spectrum of disease severity from mild, through to moderate and severe CFS/ME. All patients routinely completed the Chalder Fatigue Questionnaire. Bed bound / non-ambulatory patients were not enrolled in the study. Ninety four age and sex matched healthy controls (HC) (with exclusions of smoking or substance misuse, medication including anti-inflammatory medication or non-ambulatory subjects) were also recruited through local advertisement in the city of Liverpool and during CFS/ME recruitment sessions and served as the control group. This group also completed the Chalder Fatigue Questionniare. Sample size was determined according to power calculations to allow for a significant difference in serum 25[OH]D to be detected between the two cohorts. Individuals were excluded from the study if they smoked, had a history of substance misuse or had any underlying inflammatory based medical conditions or treatments. This study conformed to the standards set by the *Declaration of Helsinki,* and the procedures were approved by the local University of

review only

Liverpool peer review committee and Multi-Centre Research Ethics Committee. All participants gave informed written consent for their participation.

**Sample Collection**

For the analysis of vitamin D metabolites including 25[OH]$D_2$ and 25[OH]$D_3$, participants provided a venous blood sample collected from the antecubital vein into two serum separator (4.7 ml) vacutainers. Blood samples were incubated for 45 min at room temperature to induce clotting, followed by centrifugation at 1500 g for 15 min at 4°C. Serum was aspirated from the samples and stored at -80°C until analysis. All serum samples were given an anonymous coded ID number, which was sequentially generated by the study team.

**Analysis of Vitamin D**

Vitamin D metabolites 25[OH]$D_2$ and 25[OH]$D_3$ were analysed as previously described [8]. 25[OH]$D_2$ and 25[OH]$D_3$ were extracted from serum samples, following zinc sulphate protein precipitation, using Isolute C18 solid phase extraction cartridges. Potential interfering compounds were removed by initial elution with 50% methanol followed by elution of the vitamins using 10% tetrahydrofuran in acetonitrile. Dried extracts were reconstituted prior to injection into a high performance liquid chromatography tandem mass spectrometer (LC–MS/ MS) in the multiple reaction mode. The multiple reaction mode transitions ($m/z$) used were 413.2 > 395.3, 401.1 > 383.3 and, 407.5 > 107.2 for 25[OH]$D_2$ and 25[OH]$D_3$, and hexa-deuterated [OH]$D_3$, respectively. The assay was validated against published acceptance criteria (FDA 2001). Assay sensitivity was determined by the lower limit of quantification (LLOQ): 25[OH]$D_3$ = 2.5 nmol $L^{-1}$ and 25[OH]$D_2$ = 2.5 nmol $L^{-1}$. Coefficients of variation (CVs) for the assay were 10% across a working range of 2.5–625 nmol $L^{-1}$ for both 25[OH]$D_2$ and 25[OH]$D_3$.

Intra-assay precision was assessed by running quality control materials (Chromsystems Instruments and chemicals. Gräfelfing, Germany) and commercially bought material (calf serum) ten times within a single run, and separately over 15 runs for inter-assay assessment. Extraction recovery was assessed by determining the amount of vitamin $D_3/D_2$ recovered from the amount spiked prior to extraction. The percentage recovery was calculated by the measured value against the sum of endogenous value and spiking concentration. Spiked recovery is determined by adding known quantity of 25[OH]$D_3/D_2$ to serum samples with different concentrations of endogenous 25[OH]D. MassCheck calibration materials and controls are traceable against NIST 972 reference material.

For peer review only

The LC–MS/MS method used in the present study has previously been validated against other commercially available assays and is regarded as the most valid and reliable technique for the assessment of vitamin D metabolites including 25[OH]$D_2$ and 25[OH]$D_3$ [30].

**Dietary/supplement analyses**

Structured open-ended food/supplement diaries were provided to all patients and control. All participants were informed on how to complete the food record. The diary also contained a written example for reference. During a 7-day period, all consumed foods/drinks and supplements had to be reported with notification of time of consumption, estimated consumed quantity expressed as a household measure, unit or weight, specification and if present, a brand name. Food diaries were analysed using WinDiets Research Version (2010) software, written by Dr. Alan Wise, The Robert Gordon University, Aberdeen.

**Patient involvement**

The development of both the study research question and outcome measures took into careful consideration the needs and impact upon the patient cohort particularly given the highly sensitive nature of this group. Careful consideration was taken over the execution of study visits and the impact of the study protocol and sample collection. The study was designed to reduce the impact and disturbance to the patient's condition. Senior specialists in CFS/ME provided advice with regards to study design. No patients were involved in setting the research question, nor were they involved in developing plans for design or implementation of the study. No patients were asked to advise on interpretation or writing up of results. Registered patient support groups within the local area of Merseyside including CFS/ME patients and their carers provided support in the recruitment of patients into the study through the dissemination of study information and recruitment. Results will be disseminated by presentation to local and national research and patient groups.

**Statistics**

Data are presented as mean ± SD throughout. The sample distribution was determined using the Pearson's coefficient (Pc) indicating a predominantly parametric distribution. Single comparisons between two groups of normally distributed data were undertaken using independent Student's t tests. For multiple comparison analyses to examine the effect of season, one-way ANOVA followed by post hoc Tukey's LSD test was used. A P-value $<0.05$ was considered to be statistically significant. SPSS 22 was used to analyse all data. Figures were constructed with Graphpad Prism v.6.

For peer review only

## Results

Details of the study population are presented in Table 1. Epidemiological studies have reported that the prevalence of CFS/ME is significantly higher in women than in men [31] and the majority of patients with CFS/ME who took part in the present study were female (66 women and 26 men). Body mass index (BMI) analyses revealed no significant differences between CFS/ME patients and HCs(26.0 and 24.4kg/m$^2$, respectively), (Table 1). Average duration of illness in the CFS/ME patients was 36 ± 4.8 months.

Total serum 25-hydroxyvitamin D (25[OH]D) was assessed by measuring the sum of 25[OH]$D_2$ and 25[OH]$D_3$ metabolites. Circulating 25[OH]$D_2$ was undetectable in 86% and 89% of CFS/ME and HC individuals, respectively (data not shown). Undetectable concentrations of circulating 25[OH]$D_2$ in human samples has previously been reported in a number of studies [32]. Total serum 25[OH]D concentration was significantly higher (p = 0.001) in CFS/ME patients compared with healthy controls (60.2 and 47.3nmol/L respectively), (Figure 1A).

To further examine the observed difference in vitamin D status between patients with CFS/ME and HC, the effects of seasonal variation in circulating concentrations of 25[OH]D was determined. Statistical analyses revealed a significant interaction between group and season for total serum 25[OH]D (Figure 1B). Patients with CFS/ME demonstrated significantly higher (p = 0.01) total serum 25[OH]D concentrations during the autumn and winter months (Autumn/Winter) compared with HC individuals (59.1 ± 24.1 and 45.6 ± 25.9 nmol/L respectively), with no significant difference evident during the spring and summer months (Spring/Summer), (Figure 1B). The average total serum 25[OH]D concentration from HCs recruited during Spring/Summer tended to be ~30% higher (p = 0.08). Although this did not reach statistical significance in comparison to individuals recruited in Autumn/Winter (60.2 ± 22.7 and 45.6 ± 25.9 nmol/L, respectively), (Figure 1B), these data are in agreement with previous clinical studies demonstrating that vitamin D status varies seasonally, with concentrations of 25 [OH]D increasing in spring and peaking at summer [33].

The higher total serum 25[OH]D concentrations in patients with CFS/ME during the Autumn/Winter months is likely to be a result of increased vitamin D intake. To address this possibility, dietary/supplement assessment of vitamin D intake from both diet and in the form of supplements was undertaken although, due to compliance issues, these data were only provided by 21 HCs and 24 CFS/ME patients (Table 2). Dietary intake of vitamin D did not differ significantly between CFS/ME patients and HC individuals. However, more CFS/ME patients reported multivitamin supplement and/or vitamin D supplement consumption than

For peer review only

HCs. Because of the increased use of supplements by patients with CFS/ME, the cohort of CFS/ME patients showed higher ($p = 0.05$) average vitamin D intake through use of supplements compared with the cohort of HCs (4.9 ± 9.7 and 0.6 ± 1.7µg/day, respectively), (Table 2). When we limit the analysis to those individuals taking Vitamin D supplements, the average intake for HCs was 4.2µg/day compared with 14.1µg/day for CFS/ME patients. In addition, analysis revealed no differences in total serum 25[OH]D in the CFS/ME patients compared with HC individuals reporting no vitamin D supplement intake (Figure 2). Overall these findings suggest that the higher serum 25[OH]D concentrations observed in patients with CFS/ME during the autumn and winter months was likely to be due to increased vitamin D intake through use of supplements. Individuals with CFS/ME reported significantly higher levels of fatigue than the healthy control group in the Chalder Fatigue Questionnaire; a widely used and validated health questionnaire (data not shown in detail). To assess whether serum concentrations of total 25[OH]D was a contributing factor to the level of fatigue in CFS/ME, 25[OH]D values from the CFS/ME group were ordered from lowest to highest and split equally into an upper and lower range (Figure 3). The data presented show no evidence of a difference in mean or distribution of Chalder Fatigue Questionnaire Score between the two groupsand so suggest that total serum 25[OH]D concentration does not appear to be a major contributing factor to the level of fatigue in patients with CFS/ME.

**Discussion**

The present findings suggest that patients with CFS/ME do not exhibit insufficient concentrations of circulating total 25[OH]D but the values are significantly higher compared with healthy controls. The average concentration of serum total 25[OH]D in CFS/ME patients was 60.2nmol/L and was greater than the sufficient cut off of ≥ 50nmol/L set by the UK and US Institutes of Health[33]. Scores from the Chalder Questionnaire revealed no differences between CFS/ME individuals with high or low total 25[OH]D concentrations. This finding implies that vitamin D deficiency is not a major contributing factor to the pathophysiology of CFS/ME. The data from the present study further demonstrated that the average concentration of circulating total 25[OH]D in the HC group was lower (47.3 nmol/L) than the suggested sufficient concentrations (>50 nmol/L), in agreement with recent reports from the National Institute for Health and Care Excellence (NICE) that insufficient serum vitamin D concentrations are common in the UK. The present findings suggest individuals with CFS/ME have higher total 25[OH]D than healthy controls, particularly in the

For peer review only

spring/summer months, due to the consumption of vitamin D supplements. In contrast, further analysis revealed no significant differences in total 25[OH]D between CFS/ME individuals and healthy controlswho reported no supplement intake. Overall these findings imply that the higher total 25[OH]D in the CFS/ME group is likely attributed to the overall higher supplemental vitamin D intake.

Individuals with circulating 25[OH]D concentrations >50nmol/L are likely to have sufficient concentrations to maintain normal biological functions. However, concentrations between 30-50nmol/L are considered to be insufficient and <25nmol/L are likely to lead to the pathology of muscle and bone disorders such as osteomalacia and osteoporosis and to reduce overall health[34]. Previous research has been undertaken to determine changes in total 25[OH]D throughout the year as the intensity and hours of sunlight change [35]. The current study further sought to examine the changes that occur in vitamin D status throughout the year in both patients with CFS/ME and healthy control age/sex matched individuals. Seasonal variation analyses revealed that HC individuals recruited during Autumn/Winter exhibited insufficient levels of circulating 25[OH]D (<50nmol/L) with concentrations rising significantly to normal values (>50nmol/L) in individuals recruited during Spring/Summer. In contrast, there was a lack of difference in circulating 25[OH]D between Autumn/Winter and Spring/Summer in patients with CFS/ME. Data showed an ~50% reduction in vitamin D supplement intake in the patient cohort recruited during the summer months. Caution is required when interpreting these findings since the study is cross sectional and so represents different patients and these changes in supplement intake did not reach significance (data not shown) but may suggest that patients with CFS/ME reduce their supplement intake of vitamin D during the summer months. Alternatively, the patients may not have been exposed to increased sunlight during the summer months due to reduced mobility and being housebound as a result of the symptoms associated with CFS/ME. The observed suboptimal circulating 25[OH]D concentration in healthy individuals during the autumn and winter months was likely due to insufficient vitamin D intake and/or lack of adequate exposure to sunlight [13]. The higher prevalence of sufficient 25[OH]D concentrations observed in patients with CFS/ME during Autumn/Winter were associated with higher vitamin D intake through use of supplements when compared to healthy controls. Overall, these finding further suggest that it is advisable that healthy individuals consume vitamin D supplements during the autumn and winter months, although levels required for the optimal function of different tissues/organs remain unclear.

For peer review only

Patients with CFS/ME suffer from a wide range of debilitating symptoms, and dependent on these there may be reduction in exposure to the sun. The current study included patients across the disease spectrum from mild severity through to moderate and severe, although excluding bed bound/non-ambulatory patients. As a result, it is plausible to assume that there may be a subgroup of patients with CFS/ME, who may be at greater risk for deficiency. One limitation of the present work was that the majority of participants who took part in the study, including CFS/ME and HC volunteers were Caucasians who lived in the North West of England hence the findings from the present study might not reflect all CFS/ME and HC populations. It is noteworthy to mention that there is evidence that vitamin D metabolism varies in different ethnic groups[34]. Future studies comparing CFS/ME including non-ambulatory patients and HC individuals from different origin, race and disease duration are also warranted. A further limitation to the study was that only the main marker of vitamin D status, 25[OH]D was measured. It has recently been shown that there is a need to assess all of the vitamin D metabolites[35]. It may be more appropriate to measure the amount of serum free and bioavailable 25[OH]D as a predictor of vitamin D status than serum total 25[OH]D, whilst controlling for vitamin D binding protein phenotype[36]. Future studies assessing these aspects of vitamin D metabolism in CFS/ME patients are warranted.

In conclusion, patients with CFS/ME do not exhibit insufficient concentrations of circulating 25[OH]$D_3$ at any time of the year. Given that this cohort of CFS/ME patients clearly display symptoms, regardless of serum 25[OH]D concentration, these data indicate that vitamin D status may not be a major contributing factor to the pathophysiology of CFS/ME patient symptoms, although further analyses of those individuals with lower circulating 25[OH]D and more detailed investigation into vitamin D metabolism is still warranted. Current health guidelines in the UK state supplementation of vitamin D should be recommended to individuals with increased risk of deficiency and the findings from the present study suggest, in agreement with other studies, that a significant percentage healthy individuals exhibit insufficient concentrations of circulating vitamin D during the autumn and winter months. The current findings do not support the notion that vitamin D concentration contributes to the continuing symptoms of CFS/ME. However, it may be important for CFS/ME patients to supplement with vitamin D during the winter months as it is well documented that non-supplemented individuals may typically present with insufficient serum 25[OH]D during the winter season.

For peer review only

**Footnotes**

We thank Colette Foster, Emmanuel Nsutebu and Libbe Ratcliffe, the Liverpool CFS/ME Therapy Service and the Royal Liverpool Clinical Research Unit for their support.

Contributors: AM, MBB, CL, LD, and GLC conceived and planned the research. KE, GKS, MS, MF, FC, DJO and JT carried out the research, supervised by AM and MBJB and funded by grants awarded to AM, CL, LD and MBB. KE and FC performed the analysis of the intake diaries. KE, GKS and JT analysed and presented the data. KE and GKS wrote the paper. All authors revised the paper.

Funding: This study was supported by grants from the British Medical Research Council, the ME Association and The University of Liverpool.

Competing interests: All authors have completed the ICMJE uniform disclosure form at www.icmje.org/coi_disclosure.pdf (available on request from the corresponding author) and declare that: the study was supported by grants from The Medical Research Council, the ME Association and the University of Liverpool; no financial relationships with any organisations, except universities, that might have an interest in the submitted work in the previous three years; their spouses, partners, or children have no financial relationships that may be relevant to the submitted work; no other relationships or activities that could appear to have influenced the submitted work.

Ethical approval: This study conformed to the standards set by the *Declaration of Helsinki,* and the procedures were approved by the local University of Liverpool peer review committee and Multi-Centre Research Ethics Committee.

Data sharing: No additional data available.

Transparency: The corresponding author (MBB) affirms that the manuscript is an honest, accurate, and transparent account of the study being reported, no important aspects of the study have been omitted, and any discrepancies from the study as planned have been explained.

Licence for Publication:

"The Corresponding Author has the right to grant on behalf of all authors and does grant on behalf of all authors, an exclusive licence (or non exclusive for government employees) on a worldwide basis to the BMJ Publishing Group Ltd and its Licensees to permit this article (if accepted) to be published in BJO editions and any other BMJPGL products to exploit all subsidiary rights.

For peer review only

Competing Interest:

"Competing Interest: None to declare."

For peer review only

## Figures

**Figure 1. (A)** Distribution plots of individual circulating total 25[OH]D concentrations (nmol/L) assessed in patients with CFS/ME and healthy controls (HC) using LC-MS/MS. *$P$ <0.05 compared with values from the HC control **(B)** Total serum 25[OH]D concentration in patients with CFS/ME and healthy controls (HC) recruited during the Spring-Summer months (1st March – 31st August) and Autumn-Winter months (1st September – 28th February). *$P$ <0.05 compared with values from the HC group recruited during Autumn/Winter. #$P$ <0.05 compared with values from the HC control group recruited during the Spring/Summer months. Close grey circles = HC and closed black boxes = CFS/ME. Each data point refers to an individual participant. Solid black and grey bars represent the mean ± SD.

**Figure 2**. Total serum 25[OH]D concentration in patients with CFS/ME and healthy controls (HC) who reported no intake of vitamin D supplements. Each data point refers to an individual participant. Solid black and grey bars represent the mean ± SD.

For peer review only

**Figure 3**. Chalder fatigue scores in patients with CFS/ME. 25[OH]D values from the CFS/ME group were ordered from lowest to highest and split equally into an upper and lower range. Each data point refers to an individual participant. Solid black and grey bars represent the mean ± SD.

For peer review only

## Tables

**Table 1.** Baseline characteristics of study participants. Data are presented as mean ± SD unless otherwise stated.

| | HC | CFS/ME |
|---|---|---|
| **Women (n)** | 58 | 66 |
| **Men (n)** | 36 | 26 |
| **Caucasian (n)** | 89 | 91 |
| **Age (Years)** | 33 ± 10 | 40 ± 10 |
| **BMI (kg/m²)** | 24.4 ± 3.4 | 26.0 ± 4.2 |
| **Illness Duration (Months)** | N/A | 36 ± 4.8 |
| **Sample Acquisition** | 94 | 92 |

**Table 2**. Dietary/supplement assessment of average Vitamin D intake by patients with CFS/ME compared with HC individuals. Data are presented as mean ± SD.

| | HC | CFS/ME | ***P* Value** |
|---|---|---|---|
| **Average vitamin D Intake – Diet (µg/day)** | 2.9 ± 2.1 | 2.4 ± 1.6 | 0.6 |
| **Average vitamin D Intake – Supplements (µg/day)** | 0.6 ± 1.7 | 4.9 ± 9.7 | 0.05 |

For peer review only

## References


1. NICE. Chronic fatigue syndrome/myalgic encephalomyelitis (or encephalopathy): Diagnosis and management of CFS/ME in adults and children. http://guidanceniceorguk/CG53/NICEGuidance/pdf/English; 2010.
2. Jason LA, Richman JA, Rademaker AW, et al. A community-based study of chronic fatigue syndrome. Archives of internal medicine 1999;**159**(18):2129-37.
3. Nacul LC, Lacerda EM, Pheby D, et al. Prevalence of myalgic encephalomyelitis/chronic fatigue syndrome (ME/CFS) in three regions of England: a repeated cross-sectional study in primary care. BMC medicine 2011;**9**:91.
4. Johnston S, Brenu EW, Staines DR, et al. The adoption of chronic fatigue syndrome/myalgic encephalomyelitis case definitions to assess prevalence: a systematic review. Annals of Epidemiology 2013;**23**(6):371-76.
5. Littorin B, Blom P, Scholin A, et al. Lower levels of plasma 25-hydroxyvitamin D among young adults at diagnosis of autoimmune type 1 diabetes compared with control subjects: results from the nationwide Diabetes Incidence Study in Sweden (DISS). Diabetologia 2006;**49**(12):2847-52.
6. Forman JP, Curhan GC, Taylor EN. Plasma 25-Hydroxyvitamin D Levels and Risk of Incident Hypertension Among Young Women. Hypertension 2008;**52**(5):828-32.
7. Humble MB, Gustafsson S, Bejerot S. Low serum levels of 25-hydroxyvitamin D (25-OHD) among psychiatric out-patients in Sweden: Relations with season, age, ethnic origin and psychiatric diagnosis. J Steroid Biochem Mol Biol 2010;**121**(1-2):467-70.
8. Owens DJ, Webber D, Impey SG, et al. Vitamin D supplementation does not improve human skeletal muscle contractile properties in insufficient young males. Eur J Appl Physiol 2014;**114**(6):1309-20.
9. Anderson PH, Turner AG, Morris HA. Vitamin D actions to regulate calcium and skeletal homeostasis. Clinical Biochemistry 2012;**45**(12):880-86.
10. Aspray TJ, Bowring C, Fraser W, et al. National Osteoporosis Society vitamin D guideline summary. Age and ageing 2014;**43**(5):592-5.
11. Basit S. Vitamin D in health and disease: a literature review. British journal of biomedical science 2013;**70**(4):161-72.
12. Srikuea R, Zhang X, Park-Sarge O-K, et al. VDR and CYP27B1 are expressed in C2C12 cells and regenerating skeletal muscle: potential role in suppression of myoblast proliferation. American Journal of Physiology-Cell Physiology 2012;**303**(4):C396-C405.
13. Venning G. Recent developments in vitamin D deficiency and muscle weakness among elderly people. British Medical Journal 2005;**330**(7490):524-26.
14. Barker T, Schneider ED, Dixon BM, et al. Supplemental vitamin D enhances the recovery in peak isometric force shortly after intense exercise. Nutrition & Metabolism 2013;**10**.
15. Barker T, Martins TB, Hill HR, et al. Vitamin D sufficiency associates with an increase in anti-inflammatory cytokines after intense exercise in humans. Cytokine 2014;**65**(2):134-37.
16. Owens DJ, Sharples AP, Polydorou I, et al. A Systems Based Investigation into Vitamin D and Skeletal Muscle Repair, Regeneration and Hypertrophy. Am J Physiol Endocrinol Metab 2015:ajpendo 00375 2015.
17. Bischoff-Ferrari HA, Dietrich T, Orav EJ, et al. Higher 25-hydroxyvitamin D concentrations are associated with better lower-extremity function in both active and inactive persons aged >= 60 y. Am J Clin Nutr 2004;**80**(3):752-58.
18. Gerdhem P, Ringsberg K, Obrant K, et al. Association between 25-hydroxy vitamin D levels, physical activity, muscle strength and fractures in the prospective population-based OPRA Study of Elderly Women. Osteoporosis International 2005;**16**(11):1425-31.


For peer review only

19. Sinha A, Hollingsworth KG, Ball S, et al. Improving the Vitamin D Status of Vitamin D Deficient Adults Is Associated With Improved Mitochondrial Oxidative Function in Skeletal Muscle. Journal of Clinical Endocrinology & Metabolism 2013;**98**(3):E509-E13.
20. Maes M, Mihaylova L, Bosmans E. Not in the mind of neurasthenic lazybones but in the cell nucleus: patients with chronic fatigue syndrome have increased production of nuclear factor kappa beta. Neuroendocrinology Letters 2007;**28**(4):456-62.
21. Wei R, Christakos S. Mechanisms Underlying the Regulation of Innate and Adaptive Immunity by Vitamin D. Nutrients 2015;**7**(10):8251-60.
22. Whitaker CH, Malchoff CD, Felice KJ. Treatable lower motor neuron disease due to vitamin D deficiency and secondary hyperparathyroidism. Amyotrophic lateral sclerosis and other motor neuron disorders : official publication of the World Federation of Neurology, Research Group on Motor Neuron Diseases 2000;**1**(4):283-6.
23. Plum LA, DeLuca HF. Vitamin D, disease and therapeutic opportunities. Nature reviews Drug discovery 2010;**9**(12):941-55.
24. Antico A, Tampoia M, Tozzoli R, et al. Can supplementation with vitamin D reduce the risk or modify the course of autoimmune diseases? A systematic review of the literature. Autoimmunity reviews 2012;**12**(2):127-36.
25. Ziambaras K, DagogoJack S. Reversible muscle weakness in patients with vitamin D deficiency. Western Journal of Medicine 1997;**167**(6):435-39.
26. Berkovitz S, Ambler G, Jenkins M, et al. Serum 25-hydroxy Vitamin D Levels in Chronic Fatigue Syndrome: a Retrospective Survey. Int J Vitam Nutr Res 2009;**79**(4):250-54.
27. Lorusso L, Mikhaylova SV, Capelli E, et al. Immunological aspects of chronic fatigue syndrome. Autoimmun Rev 2009;**8**(4):287-91.
28. Broderick G, Fuite J, Kreitz A, et al. A formal analysis of cytokine networks in Chronic Fatigue Syndrome. Brain Behav Immun 2010;**24**(7):1209-17.
29. BACME. British Association for CFS/ME: Therapy and Symptom Management in CFS/ME. http://wwwbacmeinfo/ 2015. http://www.bacme.info/.
30. Snellman G, Melhus H, Gedeborg R, et al. Determining vitamin D status: a comparison between commercially available assays. PloS one 2010;**5**(7):e11555.
31. Reyes M, Nisenbaum R, Hoaglin DC, et al. Prevalence and incidence of chronic fatigue syndrome in Wichita, Kansas. Archives of Internal Medicine 2003;**163**(13):1530-36.
32. Hollis BW. Assessment and Interpretation of Circulating 25-Hydroxyvitamin D and 1,25-Dihydroxyvitamin D in the Clinical Environment. Rheumatic Disease Clinics of North America 2012;**38**(1):29-+.
33. Bird M-L, Hill KD, Robertson I, et al. The Association between Seasonal Variation in Vitamin D, Postural Sway, and Falls Risk: An Observational Cohort Study. Journal of aging research 2013;**2013**:751310-10.
34. Mazahery H, Camargo CA, Jr., Conlon C, et al. Vitamin D and Autism Spectrum Disorder: A Literature Review. Nutrients 2016;**8**(4):236.
35. Owens DJ, Tang JC, Bradley WJ, et al. Efficacy of High Dose Vitamin D Supplements for Elite Athletes. Medicine and science in sports and exercise 2016.
36. Johnsen MS, Grimnes G, Figenschau Y, et al. Serum free and bio-available 25-hydroxyvitamin D correlate better with bone density than serum total 25-hydroxyvitamin D. Scandinavian journal of clinical and laboratory investigation 2014;**74**(3):177-83.

For peer review only

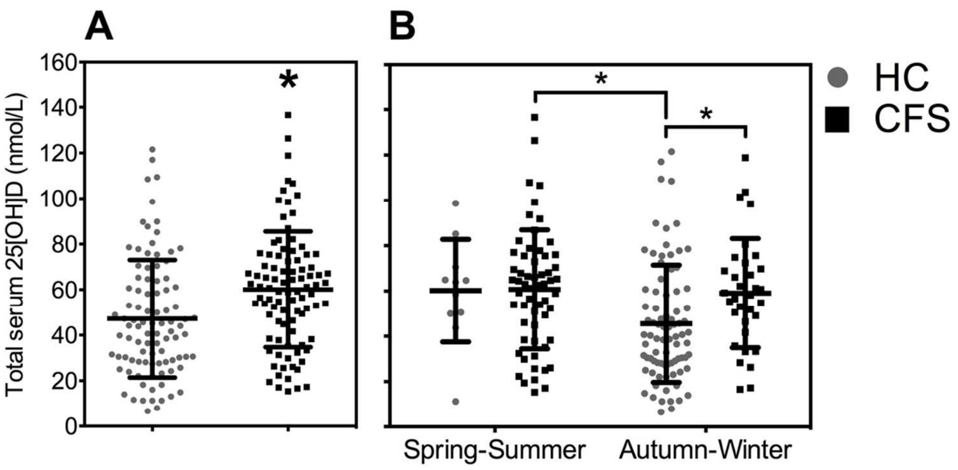


Figure 1. (A) Distribution plots of individual circulating total 25[OH]D concentrations (nmol/L) assessed in patients with CFS/ME and healthy controls (HC) using LC-MS/MS. *P <0.05 compared with values from the HC control (B) Total serum 25[OH]D concentration in patients with CFS/ME and healthy controls (HC) recruited during the Spring-Summer months (1st March – 31st August) and Autumn-Winter months (1st September – 28th February). *P <0.05 compared with values from the HC group recruited during Autumn/Winter. #P <0.05 compared with values from the HC control group recruited during the Spring/Summer months. Close grey circles = HC and closed black boxes = CFS/ME. Each data point refers to an individual participant. Solid black and grey bars represent the mean ± SD.

110x68mm (300 x 300 DPI)

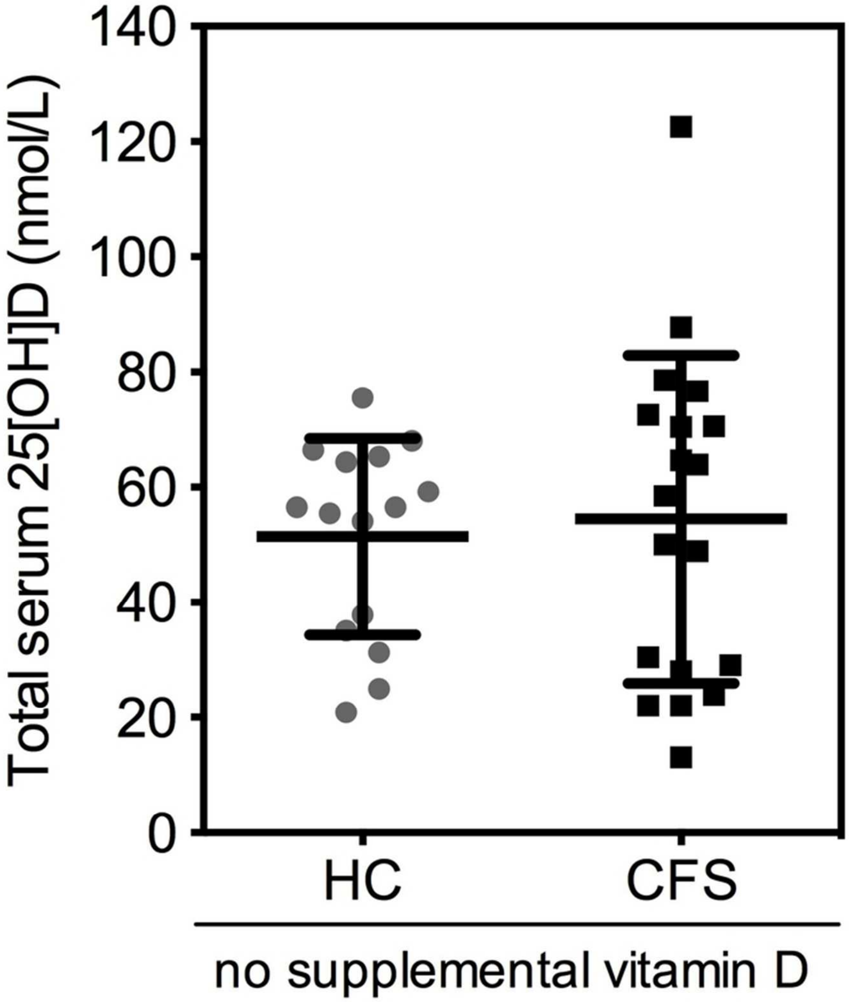


Figure 2. Total serum 25[OH]D concentration in patients with CFS/ME and healthy controls (HC) who reported no intake of vitamin D supplements. Each data point refers to an individual participant. Solid black and grey bars represent the mean ± SD.

74x78mm (300 x 300 DPI)

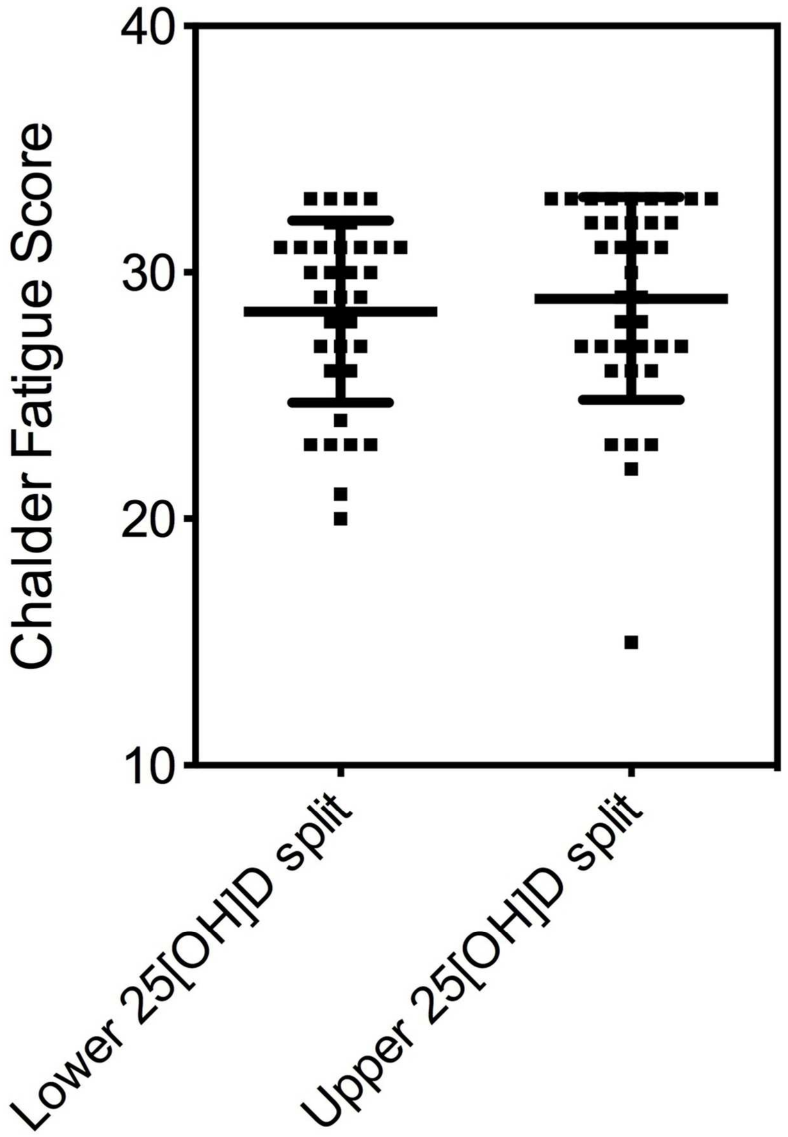


Figure 3. Chalder fatigue scores in patients with CFS/ME. 25[OH]D values from the CFS/ME group were ordered from lowest to highest and split equally into an upper and lower range. Each data point refers to an individual participant. Solid black and grey bars represent the mean ± SD.

91x134mm (300 x 300 DPI)

STROBE Statement—checklist of items that should be included in reports of observational studies

| | Item No | Recommendation | Page No |
|---|---|---|---|
| **Title and abstract** | 1 | (*a*) Indicate the study's design with a commonly used term in the title or the abstract | 1 |
| | | (*b*) Provide in the abstract an informative and balanced summary of what was done and what was found | 2 |
| **Introduction** | | | |
| Background/rationale | 2 | Explain the scientific background and rationale for the investigation being reported | 3 |
| Objectives | 3 | State specific objectives, including any prespecified hypotheses | 4 |
| **Methods** | | | |
| Study design | 4 | Present key elements of study design early in the paper | 4 |
| Setting | 5 | Describe the setting, locations, and relevant dates, including periods of recruitment, exposure, follow-up, and data collection | 4 |
| Participants | 6 | (*a*) *Cohort study*—Give the eligibility criteria, and the sources and methods of selection of participants. Describe methods of follow-up | N/A |
| | | *Case-control study*—Give the eligibility criteria, and the sources and methods of case ascertainment and control selection. Give the rationale for the choice of cases and controls | N/A |
| | | *Cross-sectional study*—Give the eligibility criteria, and the sources and methods of selection of participants | 4 |
| | | (*b*) *Cohort study*—For matched studies, give matching criteria and number of exposed and unexposed | N/A |
| | | *Case-control study*—For matched studies, give matching criteria and the number of controls per case | N/A |
| Variables | 7 | Clearly define all outcomes, exposures, predictors, potential confounders, and effect modifiers. Give diagnostic criteria, if applicable | 4 |
| Data sources/ measurement | 8* | For each variable of interest, give sources of data and details of methods of assessment (measurement). Describe comparability of assessment methods if there is more than one group | 4 & 5 |
| Bias | 9 | Describe any efforts to address potential sources of bias | 4 & 5 |
| Study size | 10 | Explain how the study size was arrived at | 4 |
| Quantitative variables | 11 | Explain how quantitative variables were handled in the analyses. If applicable, describe which groupings were chosen and why | 6 |
| Statistical methods | 12 | (*a*) Describe all statistical methods, including those used to control for confounding | 6 |
| | | (*b*) Describe any methods used to examine subgroups and interactions | 6 |
| | | (*c*) Explain how missing data were addressed | N/A |
| | | (*d*) *Cohort study*—If applicable, explain how loss to follow-up was addressed | N/A |
| | | *Case-control study*—If applicable, explain how matching of cases and controls was addressed | 4 |
| | | *Cross-sectional study*—If applicable, describe analytical methods taking account of sampling strategy | 6 |



For peer review only - http://bmjopen.bmj.com/site/about/guidelines.xhtml

For peer review only

| | | |
|---|---|---|
| | (*e*) Describe any sensitivity analyses | 6 |

Continued on next page

For peer review only



For peer review only - http://bmjopen.bmj.com/site/about/guidelines.xhtml

| | | | |
|---|---|---|---|
| **Results** | | | |
| Participants | 13* | (a) Report numbers of individuals at each stage of study—eg numbers potentially eligible, examined for eligibility, confirmed eligible, included in the study, completing follow-up, and analysed | 6 |
| | | (b) Give reasons for non-participation at each stage | N/A |
| | | (c) Consider use of a flow diagram | N/A |
| Descriptive data | 14* | (a) Give characteristics of study participants (eg demographic, clinical, social) and information on exposures and potential confounders | 6 |
| | | (b) Indicate number of participants with missing data for each variable of interest | 7 |
| | | (c) *Cohort study*—Summarise follow-up time (eg, average and total amount) | N/A |
| Outcome data | 15* | *Cohort study*—Report numbers of outcome events or summary measures over time | |
| | | *Case-control study*—Report numbers in each exposure category, or summary measures of exposure | N/A |
| | | *Cross-sectional study*—Report numbers of outcome events or summary measures | 7 |
| Main results | 16 | (*a*) Give unadjusted estimates and, if applicable, confounder-adjusted estimates and their precision (eg, 95% confidence interval). Make clear which confounders were adjusted for and why they were included | 6 & 7 |
| | | (*b*) Report category boundaries when continuous variables were categorized | 7 |
| | | (*c*) If relevant, consider translating estimates of relative risk into absolute risk for a meaningful time period | N/A |
| Other analyses | 17 | Report other analyses done—eg analyses of subgroups and interactions, and sensitivity analyses | 7 |
| **Discussion** | | | |
| Key results | 18 | Summarise key results with reference to study objectives | 8 |
| Limitations | 19 | Discuss limitations of the study, taking into account sources of potential bias or imprecision. Discuss both direction and magnitude of any potential bias | 9 |
| Interpretation | 20 | Give a cautious overall interpretation of results considering objectives, limitations, multiplicity of analyses, results from similar studies, and other relevant evidence | 8&9 |
| Generalisability | 21 | Discuss the generalisability (external validity) of the study results | 9 |
| **Other information** | | | |
| Funding | 22 | Give the source of funding and the role of the funders for the present study and, if applicable, for the original study on which the present article is based | 6 &10 |

*Give information separately for cases and controls in case-control studies and, if applicable, for exposed and unexposed groups in cohort and cross-sectional studies.

**Note:** An Explanation and Elaboration article discusses each checklist item and gives methodological background and published examples of transparent reporting. The STROBE checklist is best used in conjunction with this article (freely available on the Web sites of PLoS Medicine at http://www.plosmedicine.org/, Annals of Internal Medicine at http://www.annals.org/, and Epidemiology at http://www.epidem.com/). Information on the STROBE Initiative is available at www.strobe-statement.org.

For peer review only
